# A Novel Soluble Squalene-Hopene Cyclase and Its Application in Efficient Synthesis of Hopene

**DOI:** 10.3389/fbioe.2020.00426

**Published:** 2020-05-05

**Authors:** Zhen Liu, Yinan Zhang, Jianan Sun, Wen-Can Huang, Changhu Xue, Xiangzhao Mao

**Affiliations:** ^1^College of Food Science and Engineering, Ocean University of China, Qingdao, China; ^2^Laboratory for Marine Drugs and Bioproducts of Qingdao National Laboratory for Marine Science and Technology, Qingdao, China

**Keywords:** squalene-hopene cyclase, *Streptomyces albolongus*, squalene, hopene, biocatalysis

## Abstract

Hopene is an important precursor for synthesizing bioactive hopanoids with great commercial value. However, the chemical methods for synthesizing hopene are not efficient to date. Hopene is commonly obtained by extracting from plants or bacteria like other terpenoids, but the complicated extraction process is inefficient and unfriendly to the environment. Hopene can be biological synthesized by squalene-hopene cyclase (SHC) from squalene. However, hopene production by SHC remained at a low level until now. In this work, we found a novel SHC named OUC-SaSHC from *Streptomyces albolongus* ATCC 27414. An easy procedure for expression and purification of OUC-SaSHC was established. The conditions for OUC-SaSHC to convert squalene into hopene are optimized as in 100 mM sodium phosphate buffer (pH 7.0) containing 0.5% Tween 80, 20 mM squalene and 0.14 mg/mL OUC-SaSHC at 30°C. In the scale-up reaction with the final volume of 100 mL, the yield of squalene could be up to 99% at 36 h, and 8.07 mg/mL hopene was produced. Our work showed a great potential of OUC-SaSHC as biocatalyst on scale-up production of hopene, hence improves the SHC-catalyzing enzyme synthesis of hopene from laboratory level to application level.

## Introduction

Hopanoids are a class of pentacyclic triterpenoids which composed of hopene skeleton and a series of different side chains (Kannenberg and Poralla, 1999; Schmerk et al., 2011). These natural products play an important role in stabilizing the structure of the bacterial membrane just like the effect of sterols in eukaryote (Sáenz et al., 2012; Doughty et al., 2014). Many hopanoids have been reported to display physiological activity. For example, diploterol had been shown to be toxic to mouse leukemia cells, and the bacteriohopane-32, 33, 34, 35-tetrol has been reported to be a valuable and powerful anti-inflammatory and anti-oxidation natural product (Chen et al., 1995; Moreau and Hicks, 2001). To the best of our knowledge, hopene is the common precursor in the pathways of all known hopanoids (Bradley et al., 2010), which means hopene is an important precursor for synthesizing bioactive hopanoids (Schmerk et al., 2015; Sohlenkamp and Geiger, 2016). Compared with the chemical methods to synthesize hopene, the enzyme in multiple bond-forming reaction had a high selectivity for the overall transformation (Yoder and Johnston, 2006). Besides that, due to the low content of hopene in the natural accumulating plants or microbes (Stroeve et al., 1998), the process of extraction and purification would be complicated, not to mention the waste of resources and environment pollution. Therefore, enzymatic catalysis is considered to be a not only mild but also environmental friendly method for efficiently synthesizing hopene.

**FIGURE 1:**
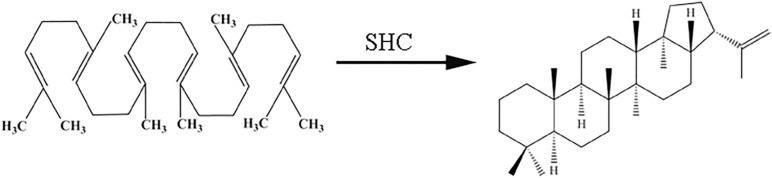
Hopene formation from squalene catalyzed by squalene-hopene cyclase.

In the biosynthetic pathway of hopene, the squalene-hopene cyclase (SHC) could catalyze the formation of hopene from its precursor squalene (Nakano et al., 2019; Figure 1). SHC is a famous enzyme for several reasons: first, the reaction of converting squalene to hopene catalyzed by SHC is the most complex already known natural one-step biochemical reaction, in which five rings and nine stereocenters were formed and thirteen covalent bond were changed (Yoder and Johnston, 2006; Abe, 2007). Second, SHCs contain QW-sequence repeats which are unique to the known triterpene cyclases (Frickey and Kannenberg, 2009). Third, all reported SHCs belong to a rare kind of membrane protein classified as “monotopic,” for SHC submerged from one side into the non-polar part of the phospholipid bilayer without protruding through it (Wendt et al., 1999). Hence, SHC and the reaction it catalyzed are investigated by many biochemists (Kannenberg and Poralla, 1999; Giner et al., 2005; Gustafsson et al., 2017; Kühnel et al., 2017; Fukuda et al., 2018; Takahashi et al., 2018; Nakano et al., 2019).

To date, several SHCs have been characterized. As early as 1986, the first prokaryotic SHC was purified from the thermophilic bacterium *Alicyclobacillus acidocaldarius* and its activity was tested *in vitro* (Seckler and Poralla, 1986). Following that, characterization and heterologous expression of it was also carried out (Ochs et al., 1990, 1992). Later, SHC from *A. acidocaldarius* became the model enzyme of this field, the structure and reaction mechanism of it have been revealed (Wendt et al., 1997; Hoshino et al., 2004; Kühnel et al., 2017; Ideno et al., 2018). In the work on SHC from *Zymomonas mobilis*, the substrate specificity of SHC was investigated. It was concluded that SHC can require many terpenoids as substrates (Seitz et al., 2012, 2013). For the SHC from *Streptomyces peucetius*, the optimal conditions of converting squalene to hopene is at pH 7.0 and 35°C (Ghimire et al., 2009). Other SHCs from *Pseudomonas mendocina*, *Bradyrhizobium japonicum*, *Methylococcus capsulatus*, *Rhodopseudomonas palustris*, and *Tetrahymena thermophile* have also been reported (Saar et al., 1991; Kleemann et al., 1994; Perzl et al., 1997; Tippelt et al., 1998; Nair and Kochupurakal, 2019).

In this work, we found a novel soluble SHC from *Streptomyces albolongus*. After heterologous expression in *Escherichia coli*, high activity of this SHC to convert squalene into hopene was detected. Furthermore, the soluble feature of our SHC enables simple Ni^2+^-NTA purification, so enough enzymes could be obtained for scale-up reaction. Our work makes the large scale production of hopene from squalene feasible.

## Materials and Methods

### Bacterial Strains and Culture Conditions

*S. albolongus* ATCC 27414 was cultivated at 28°C, pH 7.3 with shaken at 180 rpm in 50 mL medium which consisted of 1% (w/v) soluble starch, 0.2% yeast extract. *E. coli* strains were cultivated at 37°C, 220 rpm in LB medium [1% (w/v) tryptone, 0.5% (w/v) yeast extract and 1% (w/v) NaCl] or using ZYP-5052 medium [1% (w/v) tryptone, 0.5% (w/v) yeast extract] containing 100 μg/mL ampicillin and 34 μg/mL chloramphenicol (Solarbio, China) in 20°C (Liang et al., 2017).

### Sequence Analysis

Clustal × program 25 was used for multiple sequence alignment analysis and using ESPript 3.0 to compare results (Larkin et al., 2007). Neighbor-joining phylogenetic tree was constructed using the molecular evolutionary genetics analysis (MEGA) 6.0 software (Frickey and Kannenberg, 2009).

### Cloning, Heterologous Expression, and Purification of Recombinant OUC-SaSHC

A TIANamp Bacteria DNA Kit (Tiangen Biotech, Beijing, China) was used to extract the genomic DNA, which was used as a template to amplify the nucleotide sequence of *sashc* with primers (F-GGAAGATCTGTGACAGCAACCGCGGAC, R-CTAGCTAGCTGCTCCGCCTCCCGTCAG) and restriction sites is *Nhe*I and *Bgl*II. After designing and synthesizing of the primers, the gene was amplified and connected with vectors (pET-His) using Vazyme One Step Clone Kit (Nanjing, China). After transformation of vector into *E. coli* BL21(DE3) pLySs, expression of OUC-SaSHC was carried out by cultivating the strain in the ZYP-5052 medium (ZYP-5052) at 20°C, 220 rpm for 48 h. 1 mL inoculum (2%) was inoculated into 50 mL medium contained in the 250 mL Erlenmeyer flask. After culturing, cells were collected by centrifugation with 50 mL centrifuge tube at 8000 × *g* for 15 min at 4°C, and then resuspended in saline to wash. After the second centrifugation, the cells from 50 mL fermentation broth were resuspended in 10 mL buffer (20 mM Tris-HCl, pH 8.0) for disruption (amplitude 40%, 300 W, on 3 s, off 3 s for 30 min). After disrupted by sonication, the supernatant was collected after centrifugation with 50 mL centrifuge tube at 10000 × *g* for 20 min at 4°C. 50 mL supernatant was loaded onto the Ni^2+^-NTA (5 mL, Qiagen, Hilden, Germany) to purify OUC-SaSHC. Tris-HCl buffer (pH 8.0, 100 mM) containing different concentrations of imidazole (20–500 mM) have been used for protein elution. Different aliquots were determined by SDS-PAGE. Protein concentration was detected by Bradford method using coomassie brilliant blue. After purification, OUC-SaSHC was stored at −80°C until further use.

### Cyclization Assay

The crude extracts or purified OUC-SaSHC was lyophilized by vacuum freezing, then the enzyme powder was used for cyclization assay. The cyclization reaction was started by adding 0.08 mg enzyme powder into 2 mL 100 mM citrate buffer (pH 4.5) containing squalene whose final concentration is 20 mM, fill nitrogen gas to avoid substrate oxidation, then at 20°C for 16 h (Siedenburg et al., 2012).

### Product Extraction and Detection

After the catalyzed reaction was stopped (interrupted), the reaction mixture was extracted with four-fold volume of hexane. The organic phase was collected by centrifugation at 8000 × *g*, then dried by N_2_ and resolved in 300 μL of isooctane. After that, the samples were analyzed by gas chromatography (GC).

Squalene and hopene were assayed qualitatively and quantitatively by using an Agilent 6890A GC apparatus equipped with a HP-5 column (30 m × 0.25 mm × 0.25 μm) and a flame ionization detector. Two microliters of the solvent extract was applied onto the column (splitless, helium flow rate: 0.5 mL/min). The injector temperature was 250°C. The oven temperature was initially 60°C, kept constant for 3 min, then raised to 220°C at a rate of 20°C/min, kept constant for 3 min, further increased to 310°C at a rate of 6°C/min, and kept constant for 10 min. The retention time of squalene and hopene were 24.50 min and 29.70 min, respectively. Alternatively, a Shimadzu GC-MS QP 2010 system with an HP-5MS column (30 m × 0.25 mm × 0.25 μm) was used for coupled GC and mass spectrometry (MS) analysis. The condition of MS is: ion source temperature, 230°C, interface temperature, 280°C, and the temperature programming was the same as that in GC (Wu et al., 2017).

### Characterization of OUC-SaSHC

The optimal temperature was determined by conducting the reaction at 20, 30, 40, 50, and 60°C in 100 mM citrate buffer (pH 4.5) with 10 mM squalene as substrate by adding 0.08 mg enzyme powder for 16 h. The optimal pH of OUC-SaSHC was determined by carrying out the reaction at the optimal temperature for 16 h in different buffers, including citrate buffer (pH 4.0-6.0), sodium phosphate buffer (pH 6.0-8.0), Tris-HCl buffer (pH 8.0-9.0) and Na_2_CO_3_-NaHCO_3_ buffer (pH 9.0-10.0).

The effect of detergents, EDTA and metal ions on the activity of OUC-SaSHC was determined by adding 0.5% (w/v) Tween 20, Tween 60, Tween 80, or Triton X-100, 1 or 10 mM Na_2_-EDTA, CoCl_2_, KCl, NaCl, FeSO_4_, CuCl_2_, MnCl_2_, CaCl_2_, MgCl_2_, ZnCl_2_, NiCl_2_, or BaCl_2_ into the reaction mixture, then carrying out the reaction at the optimal pH and temperature for 16 h.

### Catalytic Conversion Reaction

The optimal reaction time was determined according to the productivity of hopene formation from squalene catalyzed by 0.002, 0.004, 0.006, 0.008, or 0.01 mg/mL of OUC-SaSHC in 100 mM sodium phosphate buffer (pH 7.0) containing 0.5% Tween 80, 3 mM squalene at 30°C. The optimal substrate concentration was determined by carrying out the reaction using 0.08 mg/mL OUC-SaSHC at pH 7.0, 30°C for 36 h with the substrate concentration of 0.5, 1, 5, 10, 15, 20, 25, 30, 40, 50 mM. The OUC-SaSHC concentration was optimized according to the conversion ratio of squalene in the reaction containing 20 mM squalene at pH 7.0, 30°C for 36 h by adding OUC-SaSHC with the final concentration of 0.14 mg/mL.

The scale-up experiment was conducted at the optimal conditions: 100 mM sodium phosphate buffer (pH 7.0) containing 0.5% Tween 80, 20 mM substrate, 0.14 mg/mL OUC-SaSHC at 30°C with the reaction mixture volume of 100 mL.

## Results and Discussion

### Heterologous Expression and Identification of a Novel SHC

**FIGURE 2:**
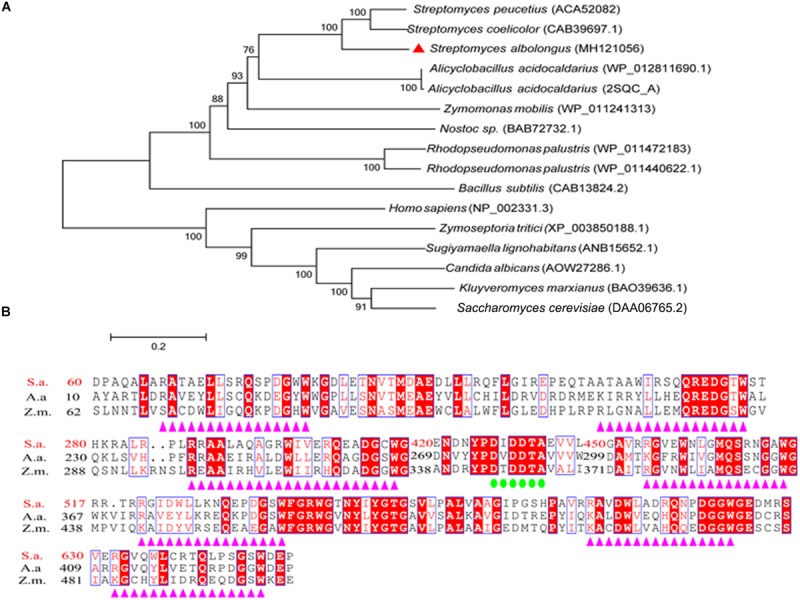
Bioinformatic analysis of squalene-hopene cyclase from *Streptomyces albolongus* ATCC 27414 (OUC-SaSHC). **(A)** Neighbor-joining phylogenetic tree analyses by MEGA 6.0 software, and OUC-SaSHC is shown as red triangles. **(B)** Multiple sequence alignments of OUC-SaSHC with squalene-hopene cyclases from Alicyclobacillus acidocaldarius and Zymomonas mobilis. The DXDDTA motif and the QW motifs are emphasized by green circles and purple triangles, respectively.

In our work on cultivation of *S. albolongus* ATCC 27414, it was found that both squalene and hopene were accumulated in the strain cells after fermentation (Data not shown). From the whole genome sequence of *S. albolongus*, one putative SHC-coding gene designated as *sashc* was found based on annotation and Basic Local Alignment Search Tool (BLAST). Gene *sashc* contains 2076 bp nucleotides and encodes putative SHC named OUC-SaSHC (GenBank accession No. MH121056) with 691 amino acids. In addition, the phylogenetic analysis result shows that OUC-SaSHC is a member of ISOPREN-C2-like super family (Figure 2A) which contains squalene-hopene cyclase and 2,3-oxidosqualene cyclase. These enzymes catalyze a cationic cyclization cascade converting linear triterpenes to fused ring compounds (Rohmer et al., 1979). Moreover, multiple sequence alignment revealed that OUC-SaSHC had one DXDDTA motif and seven QW motifs [(K/R) (G/A)X_2__–__3_(F/Y/W) (L/I/V)_3_X_3_QX_2__–__5_GXW] (Figure 2B). The function of DXDDTA motif is initiation of polycyclization reaction (Wendt, 2005) and the QW motif is necessary for reinforcement of the protein structure (Frickey and Kannenberg, 2009). These results indicated that OUC-SaSHC was a new member of ISOPREN-C2-like superfamily.

After heterologous expression of *sashc* in *E. coli* BL21(DE3) pLySs, the crude extracts of the expressing *sashc* could convert squalene to hopene (data not shown), which was verified by both GC and GC-MS with the hopene standard sample. This showed that we found a novel SHC from the actinomycete *S. albolongus*.

### OUC-SaSHC Is a Soluble Protein

**FIGURE 3:**
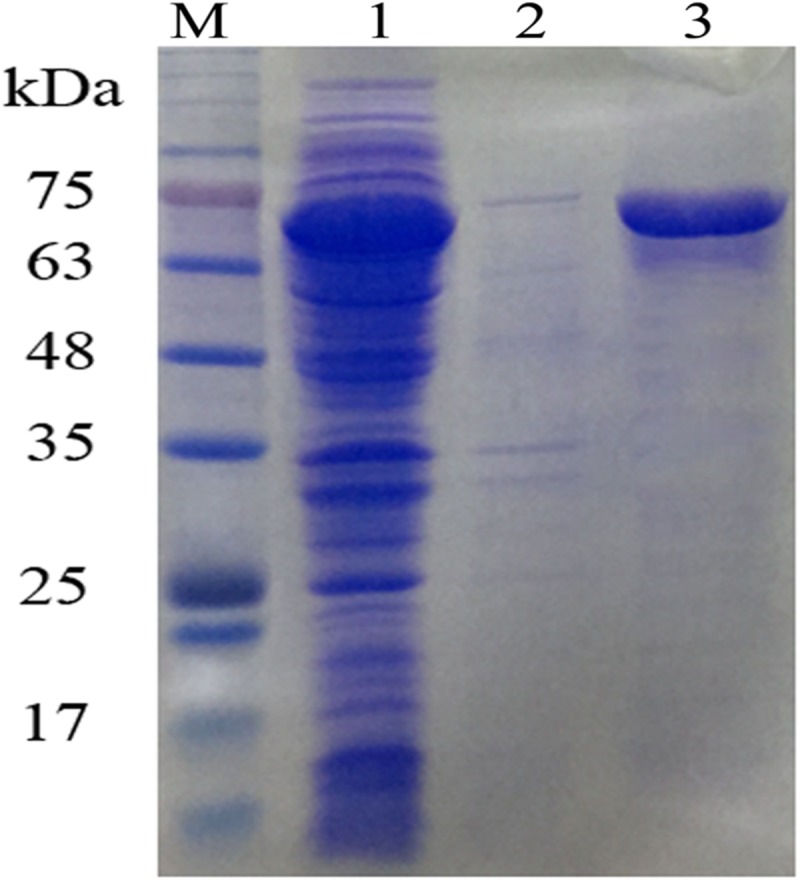
SDS-PAGE analysis of OUC-SaSHC. Lanes: M, protein marker; 1, supernatant of OUC-SaSHC cell lysate; 2, pellet of OUC-SaSHC cell lysate; 3, purified OUC-SaSHC.

**FIGURE 4:**
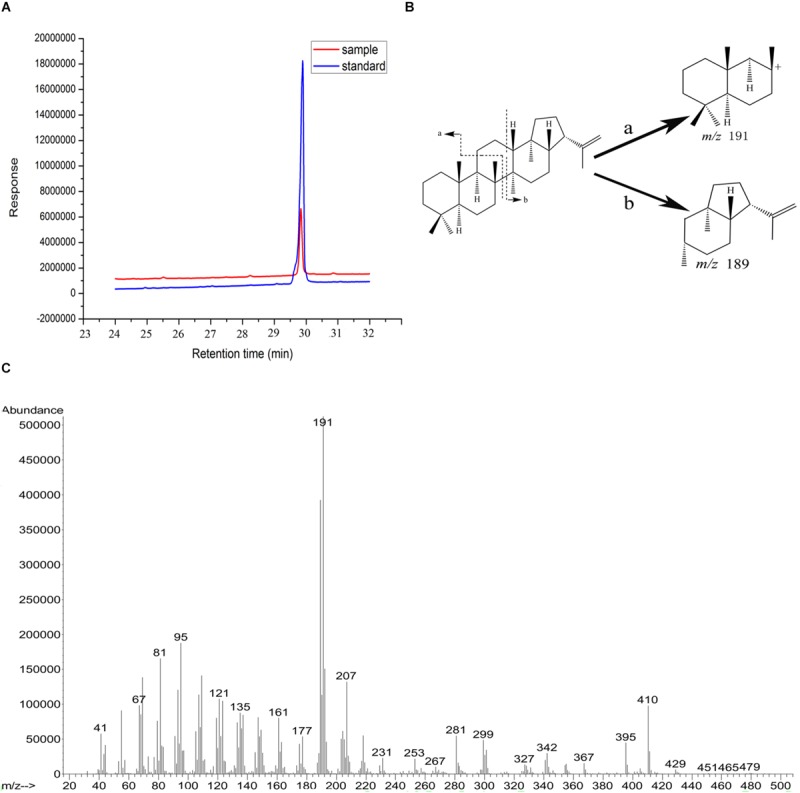
GC and GC-MS analyses of reaction products in the conversion of squalene by OUC-SaSHC. **(A)** GC results of the reaction product sample (red) and the standard sample (blue) of hopene. **(B)** The fragmentation patterns are indicative for hopene. **(C)** GC-MS profile of the reaction product sample. The molecular ion peak is at *m*/*z* = 410.

Ni^2+^-NTA sepharose column was used to purify OUC-SaSHC with the C-terminal 6 × His tag from the crude extracts, the SDS-PAGE result showed a single belt with the relative molecular mass of around 63–75 kDa, which was consistent with the theoretical value of 69 kDa (Figure 3). The purified protein was used to catalyze the cyclization of squalene. The specific activity of OUC-SaSHC was tested as 1138 U/mg, and the product was identified as hopene by GC-MS, since both the retention time (Figure 4A) and the most indicative fragments of the mass spectrum [410.4 (M^+^, 21%), 367.3 (M^+^-C, H, 9%), 341 (below 5%), 191-2 (ring C cleavage, 100%), 189.2 (ring C cleavage, 79%), *m*/*x* (%)] (Figure 4C) were consistent with the hopene standard sample, which suggests the product was indeed hopene (Figure 4B).

### Characterization of OUC-SaSHC

**FIGURE 5:**
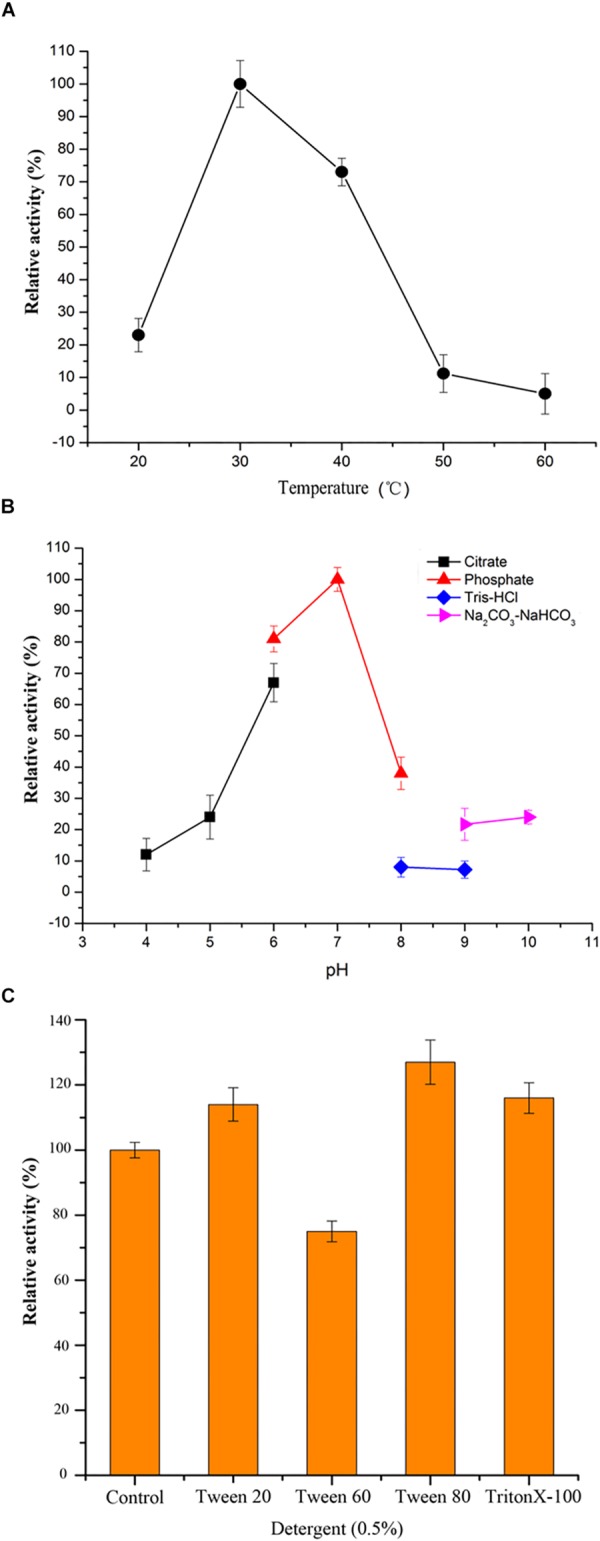
Characterization of OUC-SaSHC. **(A)** Effect of temperature on OUC-SaSHC activity. **(B)** Effect of pH on OUC-SaSHC activity. **(C)** Effect of different kinds of detergents (0.5%, w/v) on OUC-SaSHC activity. The reaction without detergents was control which activity was defined as 100%.

To find out the conditions for OUC-SaSHC exhibiting high activity, effects of temperature, pH, detergents and metal ions on the activity of OUC-SaSHC were investigated. As shown in Figure 5A, OUC-SaSHC showed highest activity at 30°C, and maintained approximately 75% of the highest activity at 40°C. When the temperature was over 50°C or below 20°C, the activity of OUC-SaSHC decreased rapidly. The optimal pH for OUC-SaSHC is 7.0. At pH 6.0, OUC-SaSHC exhibited 82% of its highest activity, while in the case of pH > 8.0 or pH < 5.0, the activity of OUC-SaSHC was less than 40% of its optimal value. This indicated that OUC-SaSHC could only exhibit high activity at neutral conditions (Figure 5B). The optimal reaction temperature for SHC from *S. peucetius* and *A. acidocaldarius* has been reported as 35 and 60°C, respectively. Similar with OUC-SaSHC, their optimal reaction conditions are in accordance with the optimum growth temperature of their original microorganisms (Ghimire et al., 2009; Siedenburg and Jendrossek, 2011).

Squalene is not only insoluble in water but also easily oxidized (Siedenburg et al., 2012). On the contrary, OUC-SaSHC was hydrophilic since it was a cytoplasmic protein. In order to promote the contact of OUC-SaSHC with squalene, 0.5% detergent was added to the reaction mixture. As shown in Figure 5C, three detergents, Tween 20, Tween 80, and Triton X-100, could increase the activity of OUC-SaSHC. When using Tween 80, the activity was increased by 29.72%.

Effect of metal ions on the activity of OUC-SaSHC is shown in Table 1. When adding EDTA, OUC-SaSHC still remained 75.26% of its normal activity, which means metal ions are not necessary for OUC-SaSHC. Many metal ions, such as Co^2+^, Na^+^, Fe^2+^, Mn^2+^, Ca^2+^, and Mg^2+^, had no obvious effects on the activity of OUC-SaSHC. Only 3 metal ions, Cu^2+^, Zn^2+^, and Ni^2+^ could inhibit OUC-SaSHC obviously. K^+^, Mn^2+^, and Ba^2+^ have a significant role in promoting OUC-SaSHC’s activity. Especially, 1 mM Mn^2+^ could increase the activity of OUC-SaSHC by 53.21%. Therefore, 1 mM Mn^2+^ was added in the reaction mixture in the application of OUC-SaSHC for hopene production in the following work.

**TABLE 1:** Effect of metal ions on OUC-SaSHC activity.

	**Relative activity (%)**	
**Ion**	**1 mM**	**10 mM**
Control	100.00 ± 1.2	100.00 ± 2.39
Co^2+^	103.26 ± 5.28	92.63 ± 3.31
K^+^	125.31 ± 0.79	123.26 ± 2.95
Na^+^	92.76 ± 2.26	82.17 ± 2.97
Fe^2+^	102.35 ± 3.78	85.79 ± 0.37
Cu^2+^	92.17 ± 5.85	63.51 ± 3.52
Mn^2+^	153.21 ± 4.53	121.03 ± 5.16
Ca^2+^	103.57 ± 3.27	99.25 ± 3.29
Mg^2+^	101.76 ± 5.23	93.16 ± 1.21
Zn^2+^	57.69 ± 0.69	52.42 ± 1.86
Ni^2+^	85.25 ± 2.72	78.29 ± 0.93
Ba^2+^	128.57 ± 3.18	103.26 ± 2.79
Na_2_-EDTA	94.17 ± 3.67	75.26 ± 1.58

### SHC Has the Potential for Large-Scale Production of Hopene

Enzymatic catalysis has not been used in the large scale preparation of hopene until now. Discovery of the soluble OUC-SaSHC will change this status, because the steady activity and easy purification steps of OUC-SaSHC will greatly decrease the cost of squalene-to-hopene reaction. Therefore, scale up of the OUC-SaSHC catalyzed hopene synthesis from squalene was investigated.

**FIGURE 6:**
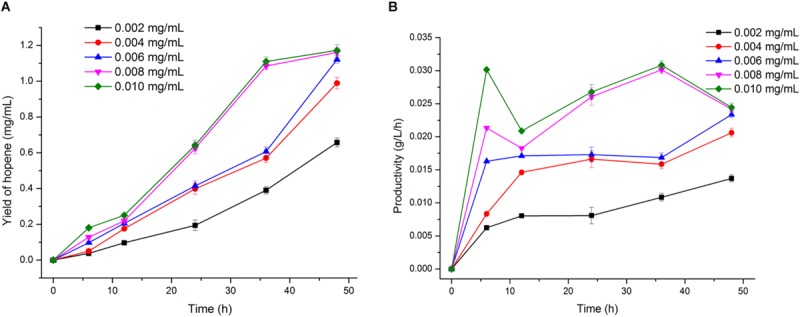
Time course of hopene yield **(A)** and productivity **(B)** at different OUC-SaSHC concentration.

Reaction time is an important factor needed to be optimized. Conversion of squalene into hopene by OUC-SaSHC was carried out in 100 mM sodium phosphate buffer (pH 7.0) containing 3 mM squalene, 0.5% Tween 80 and 1 mM MnCl_2_ at 30°C. As shown in Figure 6, when the OUC-SaSHC concentration is 0.006 mg/mL or more, hopene concentration is higher than 1.12 mg/mL at 48 h. This indicated that as long as the reaction time is enough, over 90% of squalene could be converted into hopene. Moreover, when using 0.008 mg/mL or 0.01 mg/mL OUC-SaSHC, hopene was produced with the concentration of 1.08 mg/mL or 1.11 mg/mL at 36 h, which was similar with that (1.12 mg/mL) using 0.006 mg/mL OUC-SaSHC at 48 h (Figure 6A). This suggested that reaction time could be shortened by increasing biocatalyst concentration. Furthermore, in the case of using 0.008 mg/mL or 0.01 mg/mL OUC-SaSHC, the productivity of hopene was 0.030 g/L/h or 0.031 g/L/h at 36 h, both of which decreased after 36 h (Figure 6B). Therefore, the reaction time was optimized as 36 h, with the corresponding OUC-SaSHC concentration of 0.008 mg/mL.

**FIGURE 7:**
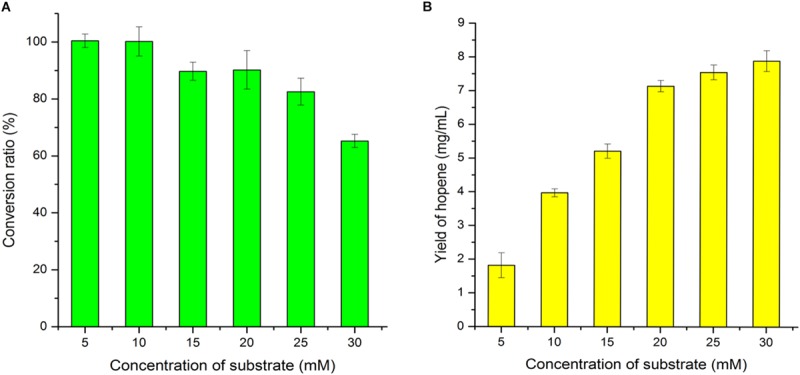
Effects of substrate concentration on squalene conversion ratio **(A)** and hopene yield **(B)**.

Substrate concentration was further optimized at optimal reaction time of 36 h. As shown in Figure 7, when the substrate concentration was 5 mM or 10 mM, 100% of the squalene was converted into hopene by 0.08 mg/mL OUC-SaSHC. With the increase of substrate concentration, the conversion ratio decreased gradually. When the substrate concentration increased from 20 mM to 25 mM, the conversion ratio decreased from 90.19 to 82.52%, and the yield of hopene increased from 7.14 to 7.54 mg/mL (Figure 7). This meant 25% of substrate increase only resulted in 5.6% of product increase, which caused waste of squalene. Therefore, 20 mM of squalene was selected as the optimal substrate concentration.

**FIGURE 8:**
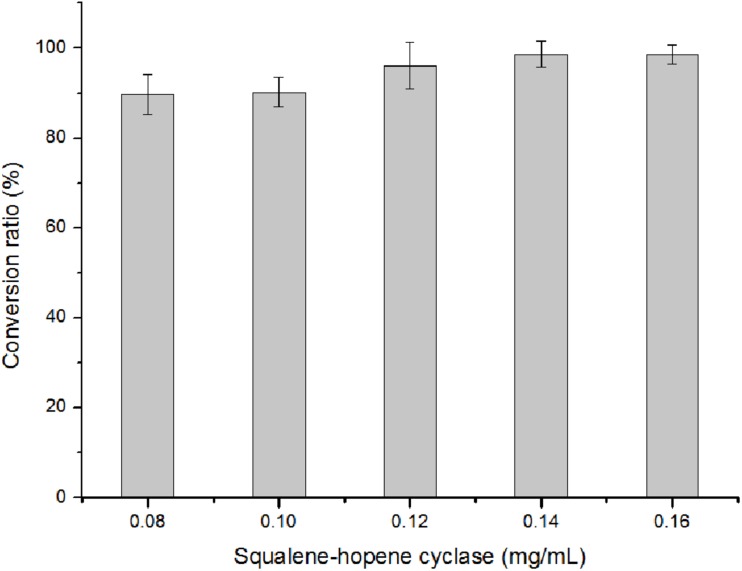
Effect of OUC-SaSHC concentration on conversion ratio of squalene.

With 20 mM of squalene as substrate, there were still 9.81% squalene remained in the reaction mixture. This suggested that further separation process was needed to obtain purified hopene. One effective way to avoid separation is to improve the conversion ratio by adding more enzymes. In Figure 8, it showed that when the OUC-SaSHC concentration was 0.14 mg/mL, the conversion ratio of squalene reached 98.72%, with 7.82 mg/mL hopene produced. It seems that the added OUC-SaSHC increased 75%, while the produced hopene only increased 9.52%. Actually, the preparation program of the soluble OUC-SaSHC is cost-effective, which means the cost of the increased 0.06 mg/mL OUC-SaSHC was much less than the value of increased 0.68 mg/mL hopene after improvement of conversion ratio from 90.19 to 98.72%.

**FIGURE 9:**
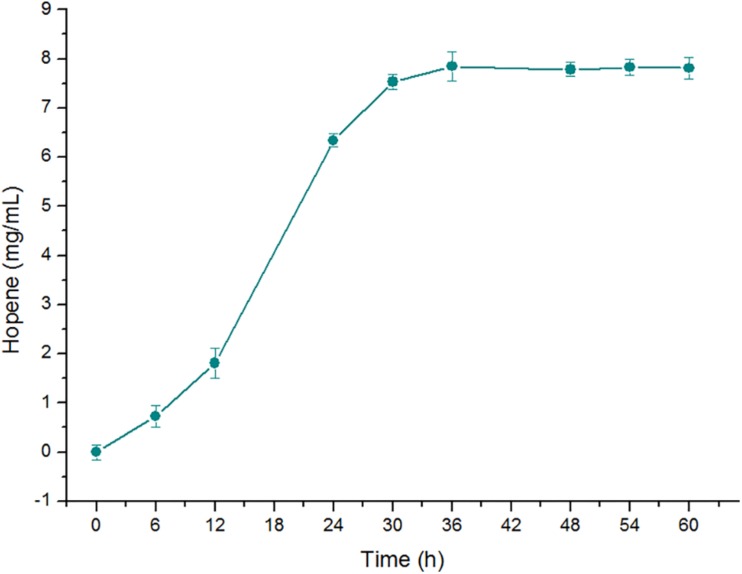
Time course of hopene concentration in the scale-up reaction in 100 mL of 100 mM sodium phosphate buffer (pH 7.0) containing 20 mM squalene, 0.14 mg/mL OUC-SaSHC, 0.5% Tween 80 and 1 mM MnCl_2_ at 30°C.

After optimization of the reaction conditions, the efficiency of OUC-SaSHC converting squalene to generate hopene has been greatly improved. The soluble properties and the high conversion ratio indicate that OUC-SaSHC has potential for large scale production of hopene. In order to further verify the feasibility of applying OUC-SaSHC for large scale production of hopene, a 50-fold scale-up reaction was conducted with the reaction volume of 100 mL. As shown in Figure 9, the reaction was carried out in 100 mM sodium phosphate buffer (pH 7.0) containing 0.5% Tween 80, 1 mM MnCl_2_, 20 mM squalene and 0.14 mg/mL OUC-SaSHC at 30°C. After reaction of 36 h, 8.07 mg/mL hopene was produced with the conversion ratio of 99%. Better data might benefit from the better mass transfer efficiency in reaction of 100 mL than that of 2 mL. This result indicated that to further improve the scale of the catalytic conversion system was feasible and expecting for producing more hopene with high purity. SHC can also be used to produce other terpenoids, such as epoxydammarane (Ideno et al., 2018) and ambrox (Eichhorn et al., 2018; List and Sun, 2018). These valuable terpenoids can also be produced at a scale-up level by using the soluble OUC-SaSHC found in this study.

## Conclusion

In summary, we discovered a novel SHC named OUC-SaSHC from *S. albolongus*. The relative molecular mass of OUC-SaSHC is 69 kDa. When using squalene as substrate, the specific activity of OUC-SaSHC is 1138 U/mg. The soluble OUC-SaSHC could be expressed and purified easily. Cost-effective preparation of OUC-SaSHC makes it possible to produce value-added hopene from squalene at a large scale. Under the optimal reaction condition, 0.81 g hopene with the purity of 99% was produced in a 100 mL catalytic conversion system. Our work shows the feasibility of large scale hopene production from squalene by the soluble OUC-SaSHC. OUC-SaSHC can also be used to large-scale produce other valuable terpenoids which can be synthesized by SHC.

## Data Availability Statement

The datasets generated for this study can be found in the GenBank Accession No. MH121056.

## Author Contributions

ZL, XM, CX conceived and designed the experiments. YZ, JS, W-CH performed the experiments. YZ, ZL analyzed the data. XM contributed reagents, materials, and analysis tools. YZ, ZL wrote the manuscript.

## Conflict of Interest

The authors declare that the research was conducted in the absence of any commercial or financial relationships that could be construed as a potential conflict of interest.
